# Case report: Molecular characterisation of adipose-tissue derived cells from a patient with ROHHAD syndrome

**DOI:** 10.3389/fped.2023.1128216

**Published:** 2023-06-30

**Authors:** Kalina M. Biernacka, Dinesh Giri, Katherine Hawton, Francisca Segers, Claire M. Perks, Julian P. Hamilton-Shield

**Affiliations:** ^1^ Cancer Endocrinology Group, Bristol Medical School, Translational Health Sciences, Southmead Hospital, Bristol, United Kingdom; ^2^ Department of Paediatric Endocrinology and Diabetes, Bristol Royal Hospital for Children, Bristol, United Kingdom; ^3^ School of Biological Sciences, University of Bristol, Bristol, United Kingdom; ^4^ Department of Translational Health Sciences, Nutrition Theme, NIHR Bristol Biomedical Research Centre, Bristol Medical School, University of Bristol, UBHT Education Centre, Bristol, United Kingdom

**Keywords:** adipocytes, ROHHAD syndrome, RNA sequencing, immune modulation, interleukin-6 and cancer

## Abstract

There have been over 100 cases of Rapid-onset obesity with hypothalamic dysfunction, hypoventilation, and autonomic dysregulation (ROHHAD) syndrome reported, but there is currently no curative treatment for children with this condition. We aimed to better characterise adipose cells from a child with ROHHAD syndrome. We isolated pre-adipocytes from a 4 year-old female patient with ROHHAD syndrome and assessed proliferation rate of these cells. We evaluated levels of DLP-Pref-1(pre-adipocyte marker) using western blotting, and concentrations of interleukin-6(IL-6) using ELISA. We performed next-generation sequencing (NGS) and bioinformatic analyses on these cells compared to tissue from an age/sex-matched control. The two most up-/down-regulated genes were validated using QPCR. We successfully isolated pre-adipocytes from a fat biopsy, by confirming the presence of Pref-1 and differentiated them to mature adipocytes. Interleukin 6, (Il-6) levels were 5.6-fold higher in ROHHAD cells compared to a control age/sex-matched biopsy. NGS revealed 25,703 differentially expressed genes (DEGs) from ROHHAD cells vs. control of which 2,237 genes were significantly altered. The 20 most significantly up/down-regulated genes were selected for discussion. This paper describes the first transcriptomic analysis of adipose cells from a child with ROHHAD vs. normal control adipose tissue as a first step in identifying targetable pathways/mechanisms underlying this condition with novel therapeutic interventions.

## 1. Introduction

Rapid-onset obesity with hypothalamic dysfunction, central hypoventilation, and autonomic dysregulation (ROHHAD Syndrome) was first described in 1965 (1), with just over 100 cases reported worldwide (2). The condition is characterised by a rapid and profound weight gain, central hypoventilation, and autonomic dysfunction (3–5). This condition has variable clinical presentation with regards to the severity and timing. Dramatic weight gain with hyperphagia in a previously well child, typically between 1.5 and 7 years of age, is often the first sign of hypothalamic dysfunction (4). This, along with at least one other clinical feature such as hyperprolactinemia, central hypothyroidism, disordered water balance, abnormal growth hormone (GH) response, adrenocortical insufficiency, or disorders of puberty is required to substantiate the evidence of hypothalamic dysfunction. The other major criterion for diagnosis is central hypoventilation caused by autonomic nervous system dysfunction. The other features of autonomic dysfunction include temperature instability, blood pressure dysregulation, arrhythmias, excessive sweating and gastrointestinal disturbances (4).

Furthermore, approximately 50% of the patients will develop tumours of neural crest origin, most commonly ganglioneuromas or ganglioneuroblastomas (4–6). The condition is often fatal, with mortality as high as 60% (4, 5, 7). Deaths are from acute cardio-respiratory arrest likely owing to a combination of peripheral and central hypoventilation (8).

The underlying pathophysiology remains undetermined, but genetic, epigenetic, and whole-body immune/inflammatory dysregulation have been suggested. Based on the disease presentation, some candidate genes have been suggested, but not subsequently associated specifically with ROHHAD and tend to be used to differentiate ROHHAD from similarly presenting conditions [reviewed in (9)]. For example, childhood obesity, hypoventilation and autonomic dysfunction are also observed in those with Prader-Willi syndrome, but Barclay et al. 2008 when assessing Prader-Willi syndrome candidate genes, were unable to identify any genetic associations with patients with ROHHAD (10). Mutation of the paired-like homeobox 2B gene (PHOX2B) is a defining factor in patients with congenital central hypoventilation syndrome (CCHS), but not in those with ROHHAD (11). Notably, there has been a case report of two affected siblings from one family (12) which may implicate a genetic/epigenetic component although one sibling had an atypical presentation. There is some evidence to suggest that ROHHAD may have an autoimmune aetiology (13–16) and clinical reports demonstrate variable response to immunomodulation (17, 18). Despite such observations and reports, currently, there is no definitive and curative, care pathway for children with this condition. With the global rise in childhood obesity, it is important for this condition to be recognized early to lessen the impact of the associated co-morbidities. This paper describes the first transcriptomic analysis of adipose cells from a child with ROHHAD compared to normal control adipose tissue from an age and sex matched child as a first step in identifying mechanisms underlying disease.

## 2. Materials and methods

### 2.1. Patient selection

Our female patient, now 5 years of age, exceeded the 99.6th centile at 4 years with weight, and body mass index at 27 kg and 26.4 kg/m^2^ (BMI-SDS +3.1) respectively following a period of rapid weight gain. At the age of 4 years and 8 months the patient was hospitalised after respiratory arrest and admitted to paediatric intensive care, requiring intubation and ventilation for 3 days. A sleep study showed both central and peripheral apnoea (apnoea hypopnoea index 22.0/hr) and elevated desaturation index with episodes of nocturnal hypoventilation. She was treated with bi-level positive airways pressure (BiPAP) and was put on nocturnal non-invasive ventilation, after which her oximetric parameters returned to normal. Before hospital admission the patient had episodes of snoring while sleeping, excessive drinking and bedwetting. Previous calorie restriction and increase in physical activity had little impact on weight gain. She was subsequently diagnosed with hyperprolactinaemia, central diabetes insipidus and growth hormone deficiency. Aged 8 years at last review, the patient maintains a BMI of 22.4 kg/m^2^(BMI SDS +1.94) requires nocturnal BiPAP, desmopressin and growth hormone therapy but interestingly her prolactin level has normalised and annual tumour screening with whole body MRI scanning, and catecholamine estimations have been normal.

Consent: The patient's parents provided consent to obtain a sample of adipose tissue from the child's left lower flank at the time of a longline insertion under general anaesthetic, as her condition is little understood. They have separately provided consent to this publication. The “control” sample was from a young child admitted to the children's hospital for elective surgery at which time a study was being conducted taking subcutaneous and visceral fat samples for studies relating to childhood obesity. This child was of normal weight BMI SDS of +0.14.9 The study was approved by the NRES Committee Southwest-Exeter (REC reference: 14/SW/0109).

### 2.2. Isolation and differentiation of pre-adipocytes from a biopsy from a patient with ROHHAD syndrome

In addition to adipocytes, there are other cell types found in adipose tissue, including endothelial, blood and immune cells, fibroblasts, pericytes, preadipocytes and macrophages (19) from which the pre-adipocytes were isolated and differentiated as performed previously (20).

### 2.3. Cell proliferation assay

Cell proliferation assays were performed as described previously (20). In brief, preadipocytes (passage 0 to 6) were seeded at a density of 0.3 × 106 per T175 flask and cultured in DMEM F12 (Thermo Fisher, Cat# 11330032) supplemented with 1% Penicillin and Streptomycin (Thermo Fisher Cat# 15140122), 1% L-Glutamine (Thermo Fisher, Cat# 25030149), 20% heated-inactivated foetal bovine serum (FBS). Cell proliferation was assessed after each passaging (around 7 days) by manual cell counting using trypan blue exclusion. All experiments were performed before confluency and contact inhibition occurred.

### 2.4. Cell counting

Trypan Blue dye exclusion assay was performed to assess cell proliferation as described previously (21).

### 2.5. Cell doubling time calculation

Cell doubling time was assessed for each passage. Cells were seeded at the starting density (**Xb)** and cultured over the number of days (**T in hours**). Once cells achieved around 70% confluency, they were trypsinized and the end cell number **(Xe)** was noted.

Doubling time was calculated using the formula: Doubling time = [T*(nl2)]/ [ln(**Xe/Xb**)].

### 2.6. Cytological image analysis

Cells were imaged using a Zeiss Axio phase contrast microscope (Jena, Germany) and a Nikon 990 Coolpix camera. Histological analyses of fixed cells were made using an Olympus BX40 fluorescence microscope (Olympus America, Inc.). Image acquisitions were calibrated with a 1 mm objective micrometer and were controlled and analyzed using Image Pro Plus 4.0 software from Media Cybernetics (Silver Spring, MD).

### 2.7. Protein assessment using Western blotting

Western blotting was performed as described previously (21). In brief, protein cell lysates (50ug), were subjected to SDS polyacrylamide gel electrophoresis, transferred to a nitrocellulose membrane (BioRad, Watford, UK), and immunoblotted with the following antibodies: DLK-Pref1 (*N*-18) (1:250, Santa Cruz, sc-8623, Dallas, Texas, USA) and GAPDH (1:5000, Merck Millipore Hertfordshire, UK). After incubation with secondary antibodies conjugated to peroxidase (Sigma, St. Louis, MO, USA), proteins were detected with a Clarity ECL substrate (BioRad, Watford, UK) using BioRad Chemidoc XRS + system and quantified using Image software.

### 2.8. Interleukin-6 (Il-6) ELISA

IL-6 is a pleiotropic cytokine that plays an important but complex role in in regulating the function of adipose tissue and is often measured as a marker of inflammation [reviewed in (22, 23)].

Levels of IL-6 in conditioned media were measured using a DuoSet ELISA kit (DY206, R&D Systems, Minnesota, USA) according to the manufacturer's instructions. Briefly, a 96-well plate was coated with mouse anti-human IL-6 capture antibody overnight, washed with 0.05% tween in PBS, blocked with 1% BSA in PBS for 1 h and washed again. Next, 100ul of standards and samples were incubated at room temperature for 2 hrs. After washing the wells, biotinylated goat anti-human IL-6 detection antibody was added and incubated for a further 2 hrs. Following an additional washing step, 100 ul of streptavidin-HRP solution was added for 20 mins and protected from direct light. The wells were washed and incubated with 100 ul of substrate solution [1:1 mixture of H2O2 and Tetramethylbenzidine (R&D Systems, Catalog # DY999)] for 20 mins in darkness followed by the addition of 50ul of 2N H2SO4 (R&D Systems, Catalog # DY994). Optical density was determined using a spectrophotometer at 450 nm (iMark™ Microplate Absorbance Reader, California, USA) with wavelength correction at 570 nm.

### 2.9. RNA sequencing of pre-adipocytes from a patient with ROHHAD syndrome

Total RNA was isolated and prepared from 3 independent passages of pre-adipocytes from the patient with ROHHAD syndrome and from 3 independent passages of pre-adipocytes from a sex and age matched control as described previously (24) using the Trizol-chloroform method (Thermo Fisher). A total volume of 14ul of total RNA of varying concentrations was provided to the Bristol Genomics Facility (University of Bristol, UK) for quantification using the Nanodrop ND-1,000 instrument (Nanodrop Technologies Inc.) and quality assessment using the Agilent RNA screentape assay on the Agilent 2,200 TapeStation instrument (Agilent, Inc). RNA Integrity values ranged from 4.9–6 confirming partial degradation of RNA. A total amount of 250 ng of total RNA for each of the six samples was taken directly into the Illumina TruSeq Stranded Total RNA kit (Illumina®) and the protocol was followed according to manufacturer's instructions without deviation. The libraries were barcoded with IDT For Illumina TruSeq RNA UD Indexes (Illumina®). Final libraries were quantified using the Thermofisher High Sensitivity dsDNA Qubit assay (Thermofisher Scientific, Inc., Waltham, MA) and validated using the TapeStation (Agilent) High Sensitivity DNA1000 screentape assay on the Agilent 2,200 TapeStation instrument. The libraries were normalised to 4nM, pooled equimolarly, and diluted to 1.2 pM for sequencing on the Illumina NextSeq500 instrument and NextSeq Control Software Version 2.2.04. An Illumina Version 2.5, Mid-Output, 150-cycle sequencing kit (Illumina Inc, San Diego, CA) was used, yielding approximately 22.2 million paired-end 2 × 75 base pair reads per sample. A spike-in of a PhiX control library Version 3 (Illumina Inc., San Diego, CA) at 2 percent was added to the sequencing run as a quality control library for the run. Primary data analysis was completed by on-board real-time analysis software (RTA Version 2.4.11). Data visualisation and QC metrics were assessed using Illumina BaseSpace Sequence Hub (basespace.illumina.com).

### 2.10. RNA-Sequencing data generation and pre-processing

All raw reads were pre-processed for a variety of quality metrics, adaptor removal, and size selection using the FASTQC toolkit to generate high quality plots for all read libraries (25). We adopted a phred30 quality cutoff (99.9% base call accuracy). RNAseq alignment and data analysis were all performed in house using our high-performance computer; “Aegaeon”. Our pipeline makes use of bash and python scripting to accept RNAseq post-trimmed data as input, before ultimately producing output tables of differentially expressed transcripts. Paired-end (2 × 75 bp) raw input data is initially trimmed for any remaining adaptors using the BBDuk suite of tools (26). Curated reads were then aligned with STAR to the latest iteration of the human genome (GRCh38) (27),. FeatureCounts is used to generate read counts, using the 11.1 annotation for reference (28). Our pipeline then uses DESeq2 from the R Bioconductor package to normalise the FeatureCounts generated count matrix and call differential gene expression (DGE) via the Wald test function to compare each of the experimental groups (29). Benjamini-Hochberg multiple test correction was used to produce the final *P*-Adjusted values that could be used for downstream data mining. For the hundred differentially expressed genes with the lowest adjusted *p*-values a heatmap of z-scores was created using the ComplexHeatmap package v2.12.1 (Gu 2022) in R v4.2.0 (R Core Team 2022). Samples and genes were hierarchically clustered by euclidean distances (30). We used g:Profiler (https://biit.cs.ut.ee/gprofiler/) software for pathway enrichment analysis (31).

### 2.11. Validation of RNA sequencing data using real-time quantitative PCR

The 4 most significant hits from the RNA sequencing analysis were subjected to validation using real-time quantitative PCR as described previously (24). qPCR was performed using SYBR Green (Applied Biosystems) on a StepOne RealTime PCR machine (Thermo Fisher Scientific). Primer sequences were as follows: Somatomedin B And Thrombospondin Type 1 Domain Containing (SBSPON) F- 5′-GTGTTTCTGCGACCAAGCCTG-3′, R-5′-GGTTGTAGGCTTGCACTGGTCT-3′, carboxypeptidase X, M14 Family Member 2(CPXM2) F- 5′-GTGCGCGGGAAGAAATGAC-3′, R-5′-CCTCCCTTGAGTGATGACACC-3′, Ribosomal Protein S4 Y-Linked 1(RPS4Y1) F-5′-TCTTCCGTCGCAGAGTTTCG-3′, R-5′-TGAGGAAGACGATCAGAGGAA-3′, Tubulin Beta Class I(TUBB) F- 5′-CCTTGCAGCTGGAGAGAATC-3′, R-5′-CTGTCGGGTTGAAAGAGAGC-3′ (Sigma-Aldrich). Quantification of product was conducted using the quantitation comparative CT function within StepOne software (ThermoFisher Scientific). Relative expression was calculated using the Pfaffl method (32).

## 3. Results

### 3.1. Characterisation of pre-adipocytes from a patient with ROHHAD

**Figure 1:**
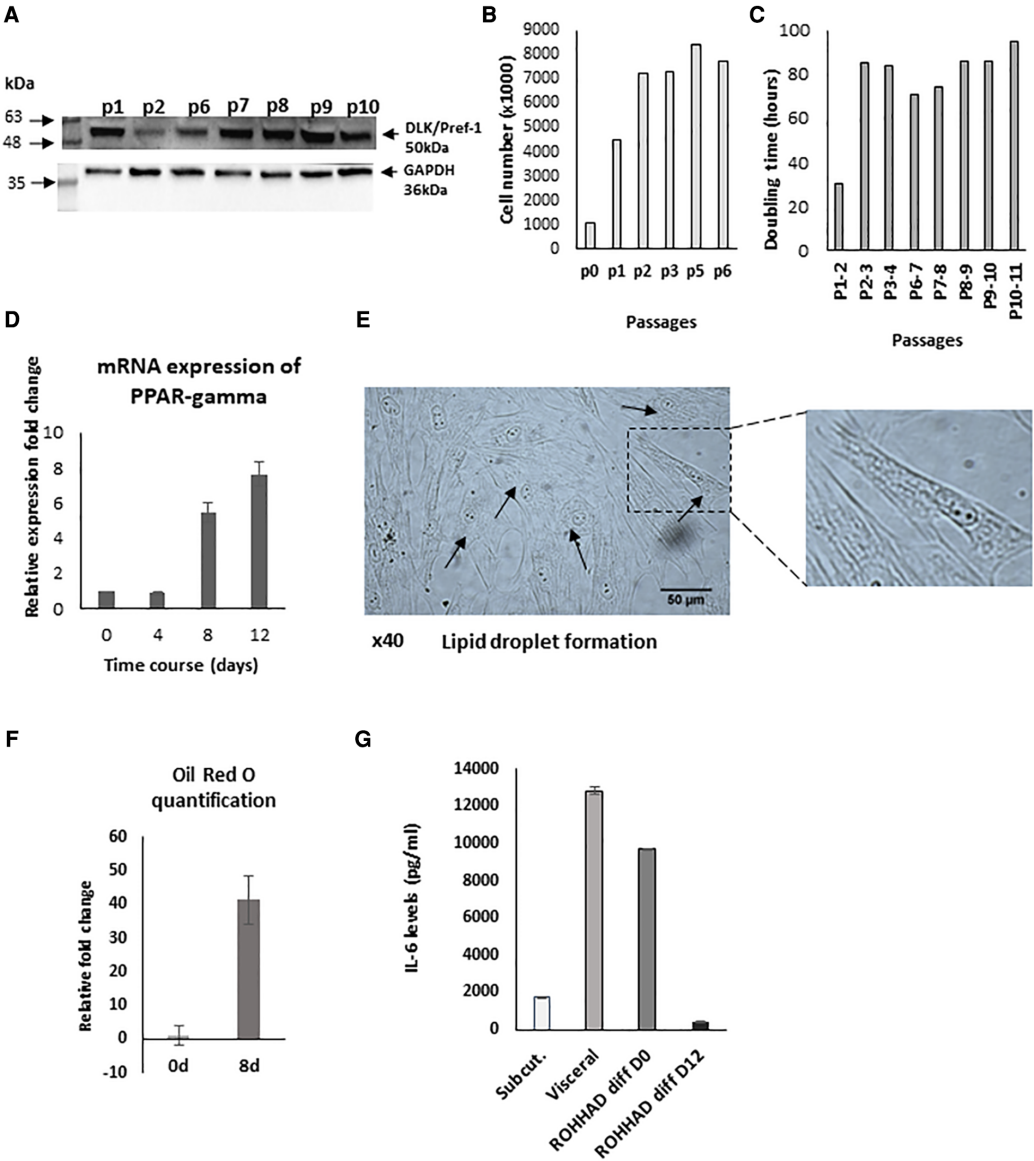
Characterisation of pre-adipocytes from a patient with ROHHAD. (**A**) Western blot indicating the presence of the preadipocyte marker, preadipocyte factor 1 (Pref-1) also known as delta-like homolog 1 (DLK1). (**B**) Graph shows cell number and doubling time (**C**) of pre-adipocytes. *P* = passage number of cells. (**D**) shows relative mRNA expression of PPAR-gamma, using GAPDH as internal control. PPAR-gamma is a marker of pre-post differentiation of adipocytes. Preadipocytes were induced to differentiate in basic differentiation media for 16 days. At days 8 and 12, expression significantly (*P* < 0.001) increased compared to day 0 (*n* = 3). (**E**) shows a photomicrograph demonstrating lipid droplet formation in mature adipocytes as indicated by arrows at day 16 (x40) with a section enlarged to visualise the lipid droplets. (**F**) Graph shows quantification of Oil Red O staining at day 0 and day 8 during the differentiation process (**G**) shows IL-6 levels from subcutaneous/visceral adipose tissue-derived pre-adipocyte from an age and sex matched control and in ROHHAD pre-adipocytes during differentiation process (at day 0 and day 12).

We initially determined that pre-adipocytes had been successfully extracted from the biopsy by confirming the presence of the specific pre-adipocyte marker, preadipocyte factor 1 (Pref-1) also known as delta-like homolog 1 (DLK1) (Figure 1A). The extracted cells were initially placed in a small T25 flask. Passages 1-2 were cultured in T75 flasks and once established, from passage 3 cells were grown in T175 flasks. From assessing cell number at each passage (Figure 1B), we were able to determine the doubling time of the pre-adipocytes with increasing passage. The doubling increased approximately 3-fold at P2-P3 and this was maintained to P10-P11 (Figure 1C). To further characterise our primary cell culture model during the differentiation process, we assessed the expression of PPAR-gamma, which is highly specific for adipose tissue and known to be upregulated during adipogenesis (33). We found a significant (*P* < 0.01) increase in PPAR-gamma expression on days 8 and 12 (Figure 1D) and, also observed the formation of lipid droplets in the mature adipocytes highlighted by the arrows (Figure 1E with an enlarged section of the photomicrograph indicated). After Oil Red O staining of cells at day 0 and 8, the stain was extracted and quantified and the relative fold increase in lipid staining from D0 to D8 was 41-fold (Figure 1F). Furthermore, the ROHHAD patient's serum cytokine analysis demonstrated raised serum IL-6 levels of 25 pg/ml compared to the normal range being 0–2 pg/ml (34).

Adipocyte dysfunction is characterized by alterations in numerous factors, such as the cytokine, IL-6. We compared IL-6 levels measured in the supernatant of patient's subcutaneous cultured pre-adipocytes with those in supernatants from sub-cutaneous and visceral cultured pre-adipocytes from a normal, age/sex matched control. We found that the subcutaneous preadipocytes from the ROHHAD patient had 5.6-fold greater levels of IL-6 (9692.3 pg/ml) than those measured from the non-ROHHAD subcutaneous preadipocytes (1730 pg/ml) and by D12 of differentiation levels in the child with ROHHAD had decreased to 435 pg/ml. ROHHAD pre-adipoctyte IL-6 levels were more comparable to IL-6 levels detected in non-ROHHAD visceral pre-adipocytes (12818.6 pg/ml) (Figure 1G).

Whilst the RNAseq data indicated an increase in IL-6 gene expression (2.3-fold) in the ROHHAD pre-adipocyte cells compared with the controls, consistent with the ELISA data, the levels of IL-6 in the supernatants could were not corrected for any changes in cell number. These potential changes in IL-6 therefore need to be confirmed and interpreted with care. In addition, IL-6 expression is also increased in obese children without ROHHAD (35, 36), which means it is likely not discriminatory for ROHHAD syndrome.

**Figure 2:**
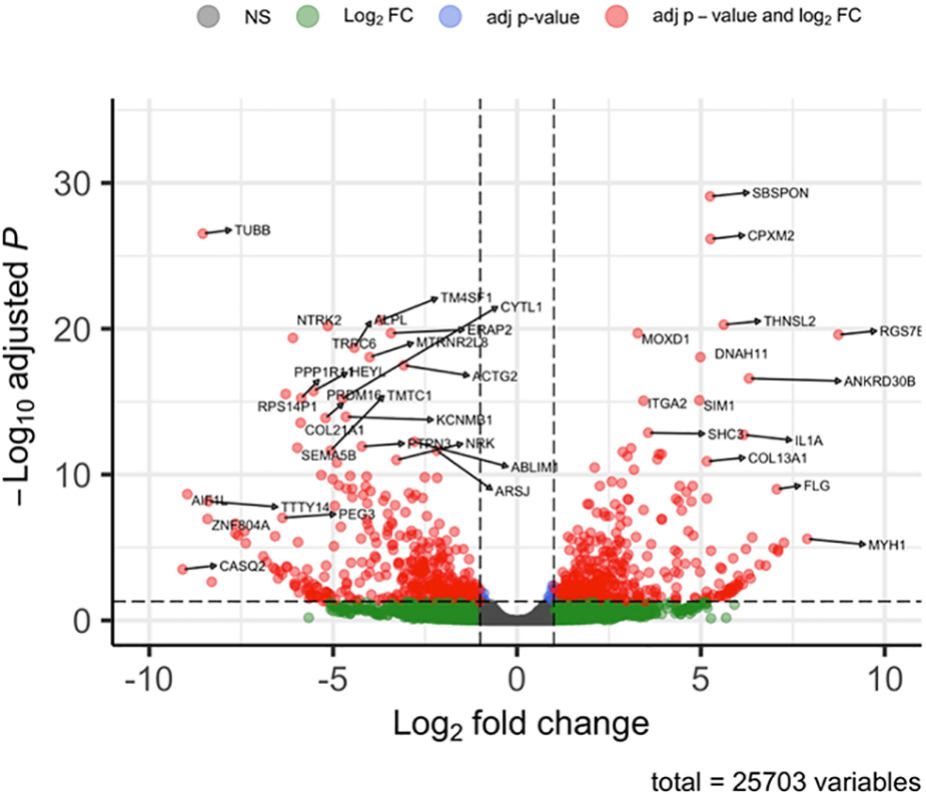
Volcano plot of overall gene expression. Red dots indicating those that were either significantly up (on the right panel of the plot) or down regulated (on the left panel of the plot) from a total of 25,703 differentially regulated genes. The remaining dots (green and black) represent genes that were not significantly changed. The representations are as follows: x-axis, log2 fold change; y-axis, -log10 of adjusted *p*-value.

**Figure 3:**
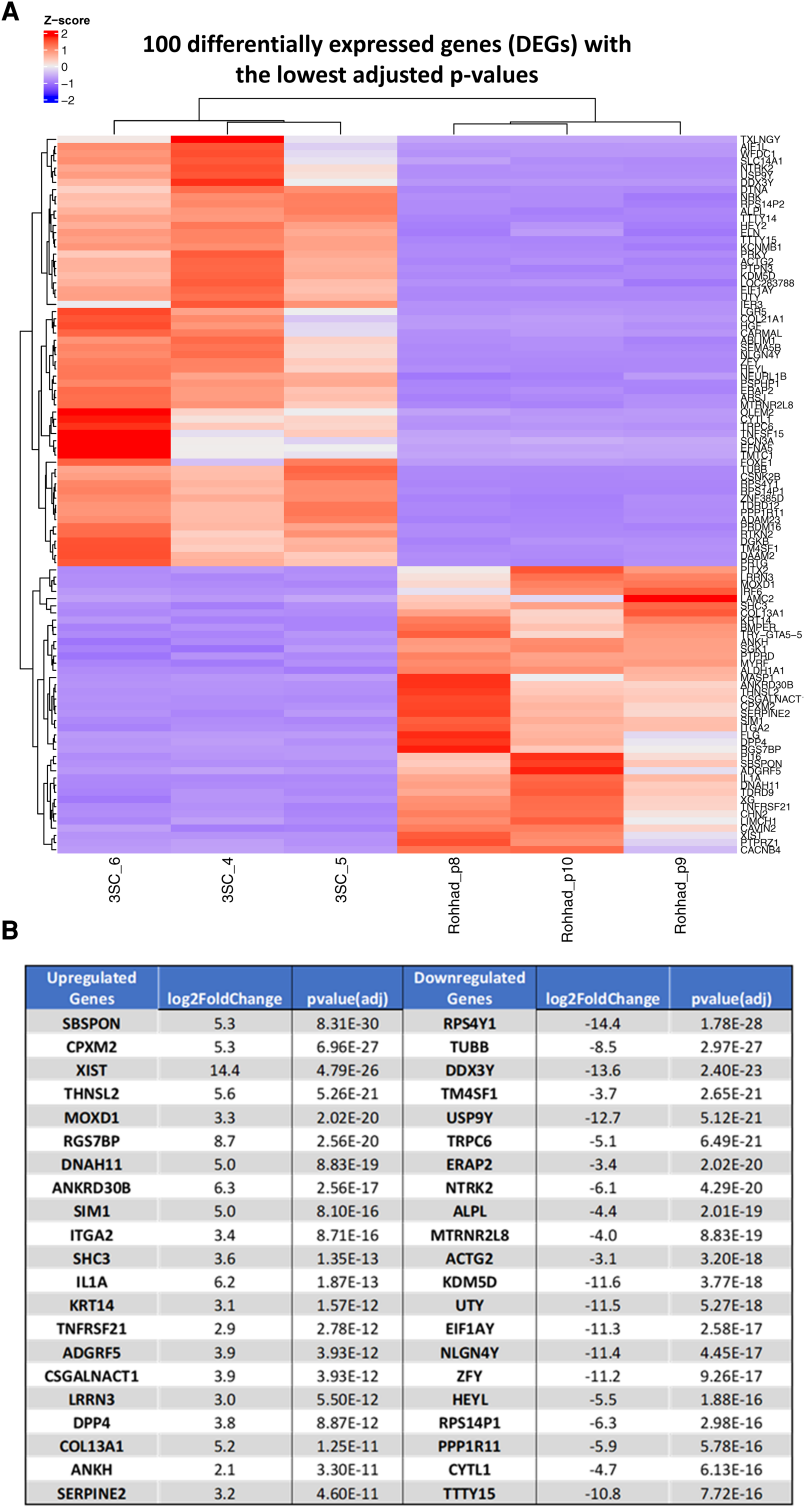
(**A**) Shows a heat map of RNA-Seq expression data showing one hundred differentially expressed genes with the lowest adjusted *p*-values a heatmap of z-scores in patient with ROHHAD syndrome vs. control samples. Pre-adipocytes from a patient (x3) with ROHHAD compared with an age and sex matched control (3SC; x3). Upregulated genes are represented in red, and downregulated genes are represented in blue. (**B**) Indicates the top 20 most significantly up or downregulated differentially regulated genes.

After analysing the transcriptomic changes between control and tissue from a patient with ROHHAD, 25,703 differentially expressed genes (DEGs) were identified in the Volcano plot (Figure 2) of which 2,237 genes were significantly differentially regulated. The corresponding 100 DEGs with lowest adjusted *p* values were shown in Figure 3A with the top 20 most significantly upregulated and downregulated genes are presented in Figure 3B.

We selected 2 genes that were each significantly up (SPSPON and CPXM2) or down regulated (TuBB and RPS4Y1) in the pre-adipocytes derived from the patient with ROHHAD compared with the control: tissue-derived subcutaneous adipocytes and using RT-q PCR, successfully validated the changes we had observed with the RNAseq data (Supplementary Figure S1).

**Figure 4:**
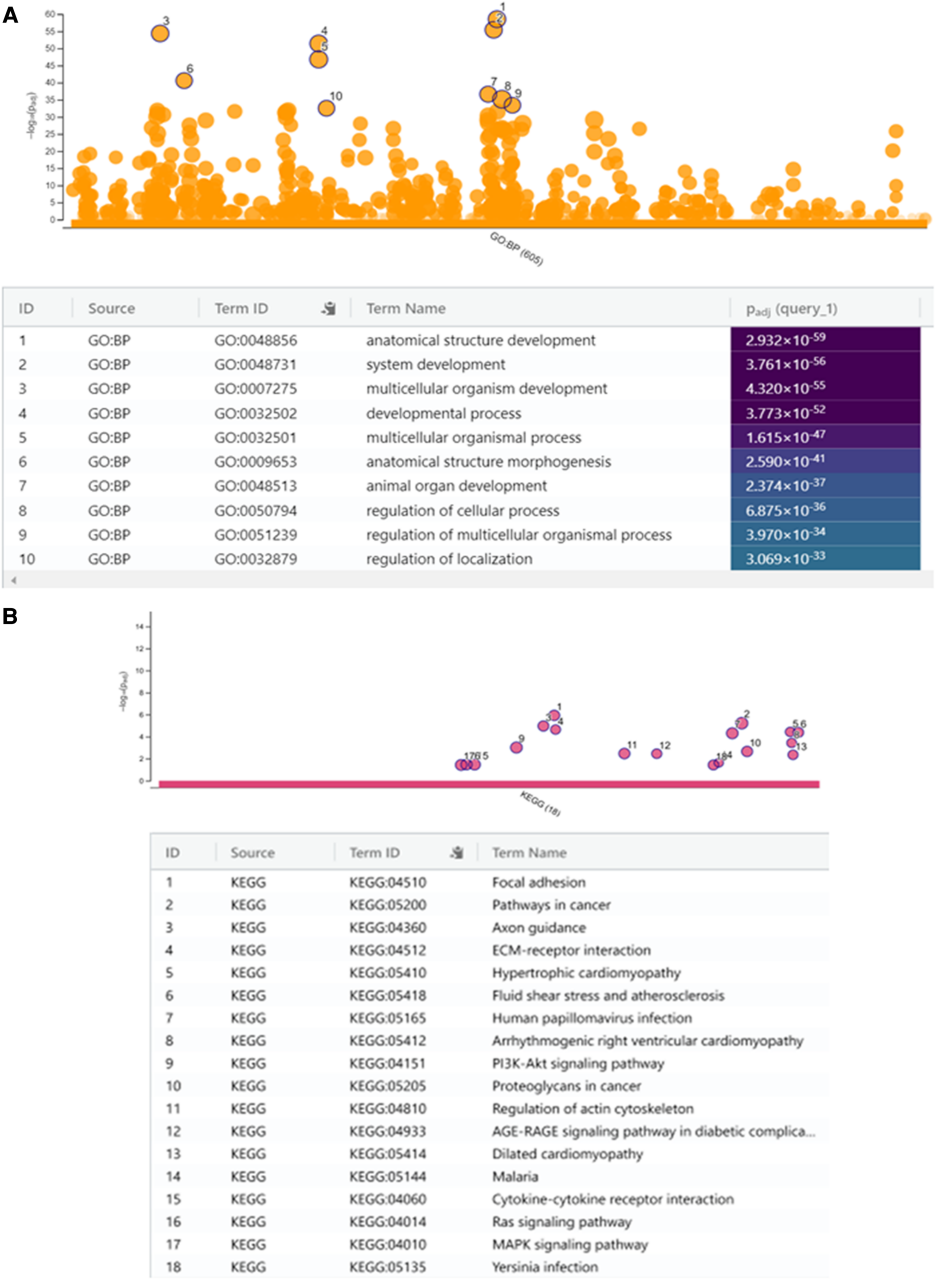
(**A**) Manhattan plot to show selected gene ontology (g:GOSt) of biological themes with enriched gene expression with the top ten most significant biological processes (BP) indicated in the table below. (**B**) KEGG analysis with 18 most significant biological pathways. KEGG analysis with enriched gene expression presented as a Manhattan plot with the 18 significant biological pathways (most significant = 1 and least = 18) indicated in the table below.

We performed biological pathway analysis using gene ontology (GO) and demonstrated enrichment of genes with pathways that were not highlighted in relation to childhood obesity alone (36, 37). These included developmental processes including “anatomical structural development” (GO: 0048856, Pcorrected = 2.932 × 10–59), “system development” (GO: 0048731, Pcorrected = 3.761 × 10–56), multicellular organism development’ (GO: 0007275, Pcorrected = 4.32 × 10–56), developmental process (GO:0032502, Pcorrected = 3.773 × 10–52) and “multicellular organismal process” (GO: 0032501, Pcorrected = 1.615 × 10–47) (Figure 4A).

We then performed a KEGG (Kyoto Encyclopedia of Genes and Genomes) analysis to uncover the most significant pathways associated with ROHHAD compared with normal tissue. The upregulated DEGs were highly associated with pathways including “focal adhesion”, “pathways in cancer”, “ECM-receptor interactions”, “axon guidance” and others (Figure 4B) of which “pathways in cancer” and, “axon guidance” were not highlighted in previous studies assessing important pathways in childhood obesity (36, 37).

## 4. Discussion

A limitation to this report lies in the comparison of our case's adipose tissue with that of a normal weight control and so the discussion will attempt to address this. Further analysis using subcutaneous adipose samples from obese children is necessary to tease out what differences are solely related to adiposity, and which are ROHHAD specific.

The clinical presentation of patients with ROHHAD is well described but the underlying aetiology of the syndrome is poorly understood. Following RNA sequencing of pre-adipocytes from a patient with ROHHAD and adipocytes from a normal control, the most significant biological pathways that we report relate to cell, tissue, organ, and system development.

The RNA sequencing data enables us to interrogate this further and try to identify key pathways and genes that may be involved and link gene changes with the key clinical characteristics associated with the condition. In this report, we shall highlight some key pathways and genes with pertinent links to ROHHAD, but clearly the data is extensive and available for further analyses. In addition, as the comparison of our case's adipose tissue is with that of a normal weight control, it is important to highlight genes that may be specific to ROHHAD syndrome and not simply attributed to the obesity associated with the disease.

We recently showed that autoimmune/inflammatory dysregulation contributed to excess adiposity in a patient with ROHHAD, as treatment with rituximab, an antibody used to combat whole body inflammation, associated with adiposity, and raised levels of proinflammatory cytokines and chemokines, significantly reduced their serum levels of IL-6 and the patient's weight (34). In keeping with this recent report, pathway analysis highlighted cytokine-cytokine receptor interaction as important as well as associated downstream signalling pathways. As cytokine-cytokine receptor interactions are also characteristic of obesity alone, this may not be specific to ROHHAD syndrome (36, 38).

We also observed differential expression of genes linked with immune regulation. For example, threonine synthase like 2 (THNSL2) (also known as SOFAT), codes for a threonine synthase-like protein that can induce IL-6 production and contribute to inflammatory conditions (39). Notably the patient with ROHHAD had significantly higher than normal levels of IL-6 in their serum and, also in their adipocytes. Other cytokines likely play a role in ROHHAD as interleukin-1 alpha (IL1A), a member of the IL-1 cytokine family, was also highly up-regulated. This gene forms part of an IL-1 cytokine gene cluster on chromosome 2 (40). Furthermore, a mediator of cytokine activity, TNF Receptor Superfamily Member 21 (TNFRSF21) (41) was also increased. The immune response and the regulation of metabolism are intricately connected, so it is perhaps unsurprising that Hawton et al. have linked adiposity with immune dysregulation in a patient with ROHHAD (27). Similarly, although clearly important characteristics of patients with ROHHAD, the upregulation of THNSL2, IL1A and TNFRSF21 have also been reported to occur in obese children without ROHHAD (36).

Pathways linked with disturbed metabolism, that are also specifically linked with obesity, were also identified, such AGE/RAGE (42) and interestingly we report a significant increase in a gene called dipepidyl peptidase 4 (DPP-4), inhibition of which in mice demonstrates a role in immune regulation, and metabolism with obesity-induced insulin resistance and inflammation (43): DDP-4 inhibitors are being used safely in current on-going clinical trials, particularly for those with type 2 diabetes (44). X inactive specific transcript (XIST) is a long-stranded non-coding RNA that was significantly upregulated in ROHHAD samples and notably a recent report has described a role for XIST in human adipocyte differentiation and in preventing high fat diet induced obesity in mice (45). Neurotrophic receptor tyrosine kinase-2 (NTRK-2) encodes for the neurotrophin receptor TrkB, that that has also previously been linked to the regulation of energy homeostasis with mutations in NTRK2 being associated with severe early onset obesity in children (46, 47). Notably, this gene may be important for ROHHAD as it was not identified as being dysregulated in normal obese children (36). This gene has an established role in tumours of neural crest origin, characteristic of ROHHAD and discussed further below.

Interestingly one of the most significantly downregulated genes in our study was Ribosomal Protein S4, Y-Linked (RPS4Y1), which was recently identified as a key regulator of fat deposition in Nandan-Yao chicken (48). CPXM2, also highly upregulated, has been associated with “fatness” in pigs (RNA-Seq) (49) and Grabowski et al., showed that CPXM2 can mediate cardiac hypertrophy and hypertension in rats (50), characteristics associated with patients with ROHHAD syndrome (8). Whether any of these genes linked with disturbed metabolism are only associated with the obesity aspect of the disease will require additional investigation.

Pathways in cancer were also observed as important from the KEGG analysis, and consistent with the development of tumours of neural crest origin as another common clinical feature of those with ROHHAD. Of all the cancer-associated genes identified including, somatomedin B and thrombospondin type 1 domain containing [SBSPON], carboxypeptidase X, M14 family member 2 [CPXM2], XIST, tubulin beta class I [TUBB], transmembrane 4 L six family member 1 [TM4SF1], to our knowledge only NTRK2 has been specifically linked to tumours of neural crest origin. Neuroblastoma arises through failure of proper sympathetic nervous system development and clinically the NTRK family are known to play a key role (51, 52). SBSPON, one of the cancer associated genes, is part of the extracellular matrix and is associated with O-glycosylation (OGT) of proteins, which is linked to nutrient availability (53, 54) and overexpression of OGT can lead to a decrease in lipolysis in adipose tissue (55). Notably, this gene may be ROHHAD-specific as it was not identified in a recent analysis of transcriptomics from the subcutaneous fat tissue of obese children (36). Whether any of the other genes highlighted, such as TUBB, that has recently been identified as a prognostic marker for ER alpha positive breast cancers (56), play a role in this syndrome is yet to be determined.

In addition to SBSPON, we identified additional upregulated genes such *as, ITGA2, FLG* and *ANKRD30B* also not identified in a recent analysis of transcriptomics from the subcutaneous fat tissue of obese children, that may relate specifically to ROHHAD (36). *ITGA2* translates to the alpha subunit of a transmembrane cell surface adhesion receptor for collagens and associated proteins and is important for many developmental processes, for example differentiation, proliferation, and migration of cells (57). The *FLG* gene encodes for pro-filaggrin, that is a major constituent of the epidermis, polymorphisms in which are associated with skin and allergic diseases (58). Finally, *ANKRD30B,* the biological function of which appears unclear, may also represent a ROHHAD-specific gene change. Studies have reported that its methylation status is important in Alzheimer's disease (59) and a rare genetic disorder called Williams syndrome (60).

We have selected a few genes from our analysis for discussion but clearly there are many others that may provide further insight into this condition. Determining key and novel differences between adipose tissue from the patient with ROHHAD compared with control, may indicate key pathways in this disease process to target in novel therapeutic interventions.

## Data Availability

The original contributions presented in the study are publicly available. This data can be found here: GEO repository, accession number GSE208445.

## Supplementary material

Supplementary Figure S1 RT q-PCR validation of four significantly regulated genes identified from the RNA Scheme 2. Most significantly up-regulated genes in ROHHAD adipocytes compared with control adipocyte biopsy (3SC) were Somatomedin B and Thrombospondin Type 1 Domain Containing (SPSPON) and Carboxypeptidase X, M14 Family Member 2 (CPXM2), where 2 most significantly down-regulated genes were ribosomal Protein S4 Y-Linked 1 (RPS4Y1) and Tubulin Beta Class I (TuBB). [B] Real-time PCR showing an increase in mRNA expression of SPSPON and CPXM2 in ROHHAD adipocytes and a decrease in mRNA expression of TuBB and RPS4Y1 adipocytes all compared to 3SC (control biopsy). Experiment repeated 3 times.
