# Supplementary figures and images for: Case report: Molecular characterisation of adipose-tissue derived cells from a patient with ROHHAD syndrome
Source: Front Pediatr. 2023 Jun 30;11:1128216. doi: 10.3389/fped.2023.1128216 (PMC10348915; doi:10.3389/fped.2023.1128216)

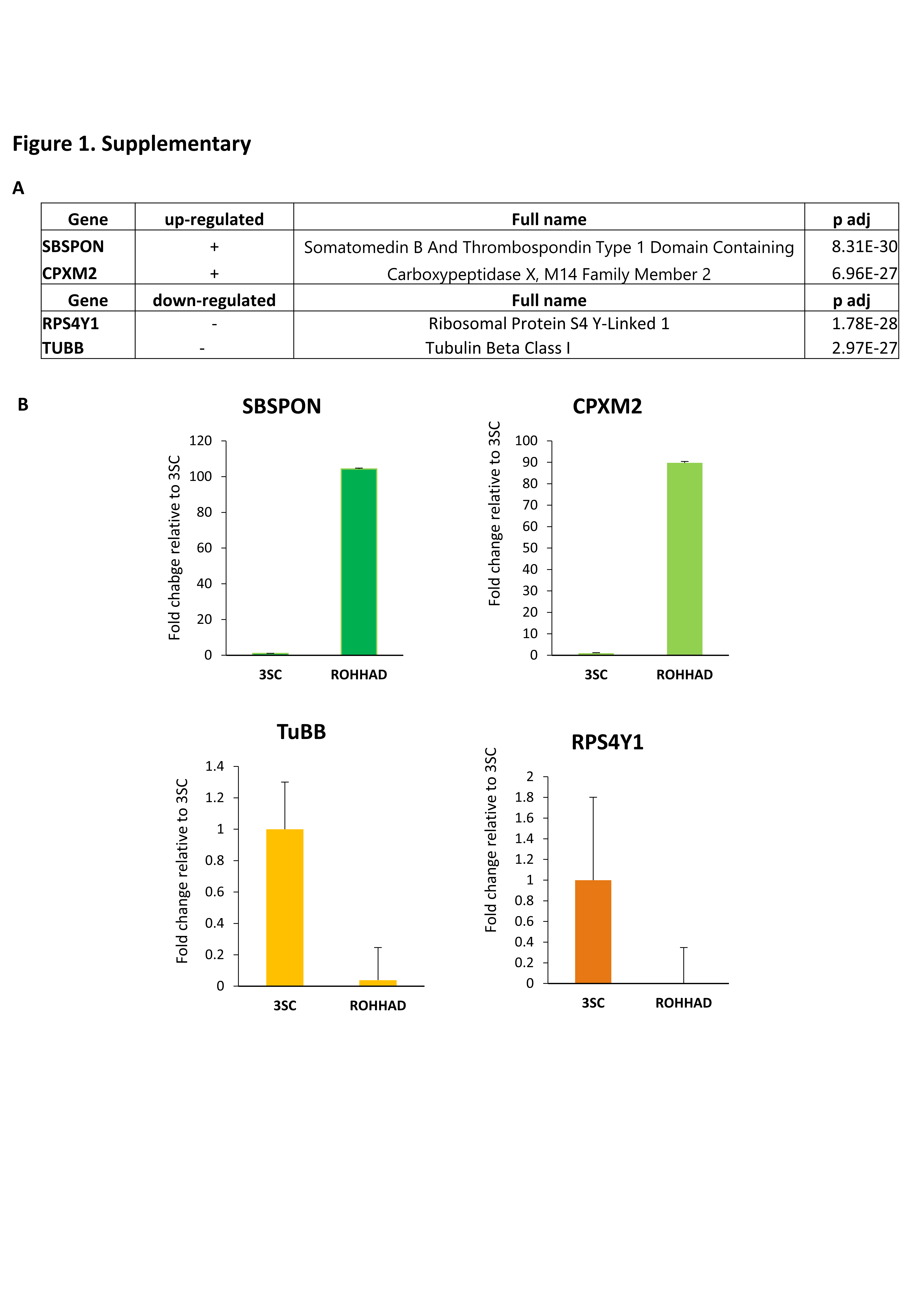

Supplement: Supplementary Figure S1 — RT q-PCR validation of four significantly regulated genes identified from the RNA Scheme 2. Most significantly up-regulated genes in ROHHAD adipocytes compared with control adipocyte biopsy (3SC) were Somatomedin B and Thrombospondin Type 1 Domain Containing (SPSPON) and Carboxypeptidase X, M14 Family Member 2 (CPXM2), where 2 most significantly down-regulated genes were ribosomal Protein S4 Y-Linked 1 (RPS4Y1) and Tubulin Beta Class I (TuBB). [B] Real-time PCR showing an increase in mRNA expression of SPSPON and CPXM2 in ROHHAD adipocytes and a decrease in mRNA expression of TuBB and RPS4Y1 adipocytes all compared to 3SC (control biopsy). Experiment repeated 3 times. [file Image1.tif]
